# The experiences of transgender and nonbinary individuals in general practice in Denmark, with a focus on ‘safer space’

**DOI:** 10.1080/02813432.2025.2599986

**Published:** 2025-12-15

**Authors:** Elliot Vexø Bennich, Ann Dorrit Guassora

**Affiliations:** Centre for General Practice: The Section of General Practice Medicine and The Research Unit for General Practice, Department of Public Health, University of Copenhagen, Copenhagen, Denmark

**Keywords:** Primary health care, general practice, transgender, nonbinary, safe space, gender identity

## Abstract

**Background:**

In Denmark, transgender and nonbinary (TNB) individuals must consult their General Practitioner (GP) to access gender-affirming healthcare (GAHC). TNB individuals report unmet health needs and higher incidences of mental health challenges compared to cisgender peers. Cultural safety, involving a ‘safe space,’ could reduce healthcare inequities.

**Objectives:**

This study aims to identify the factors, according to TNB individuals in Denmark, that create, maintain, and disrupt a ‘safe space’ in general practice.

**Method:**

Twelve semi-structured qualitative interviews with TNB individuals aged 20 to 43 were conducted, transcribed verbatim, and analysed using Systematic Text Condensation.

**Main findings:**

Participants felt unsafe with GPs due to negative experiences related to being transgender, leading to healthcare avoidance. They valued GPs who respected chosen names, showed interest in transgender care, and were upfront about limitations. A significant issue was lack of GP knowledge on transgender healthcare, often requiring participants to educate their GPs. Participants emphasised the need for doctors to avoid assumptions about patients based on gender identity. Participants did not believe that a ′safe space’ can be realised but wish for a ‘safer space’.

**Conclusion:**

Trans individuals feel unsafe when consulting their GP due to past and present negative experiences, which leads to healthcare avoidance. A ‘safer space’ encompasses; respecting chosen names, showing an interest in transgender care, listening and meeting patients on their terms. The study highlights the importance of educating GPs on respectful interactions, suggesting that adopting the concept of a ‘safer space’ as a medical term could improve healthcare for TNB individuals.

## Background

In Denmark, individuals who identify as transgender and nonbinary (TNB) must consult their General Practitioner (GP) to access primary care and obtain referrals for gender-affirming healthcare (GAHC) at one of the three Centres for Gender Identity (CKI). It is estimated that 0.5-1% of the Danish population identifies with a gender different from that assigned at birth [1]. Despite increasing visibility and Denmark’s decision in January 2017 to remove transgender identity from the list of mental disorders—thereby recognising gender incongruence as an identity issue rather than a psychiatric illness—TNB individuals in Denmark continue to experience substantial barriers and inequities within the healthcare system [1–5]. It is well-documented that they often have unmet health needs, lower self-rated health compared to their cisgender peers, and are more likely to experience mental health challenges [1–7]. Key figures from the 2019 Danish population survey, SEXUS [1], indicate that transgender individuals are more than twice as likely to report ‘bad’ or ‘very bad’ self-rated health compared to their cisgender peers. A recent study by The Danish Centre for Social Science Research (VIVE, 3) corroborates these findings. TNB individuals are more frequently treated for anxiety, depression, or stress and report higher levels of loneliness compared to cisgender individuals [1–3,6–8].

In recent years, an increasing number of international studies have documented the experiences of TNB individuals in healthcare [9–11]. Primary care has garnered significant interest as it serves as the gateway for accessing GAHC and addressing general health issues in many countries. A literature review by Holland et al. [12], including quantitative and qualitative studies on the experiences of TNB individuals in primary care, found that participants frequently encountered inadequately trained healthcare providers who lacked knowledge about trans identities and the provision of GAHC. TNB individuals reported experiencing discrimination when accessing healthcare and perceived primary care negatively on a systemic level [12].

Despite clear evidence that TNB individuals face significant health inequities, there is a gap in Danish literature regarding their encounters with general practice. A 2019 report for the Danish National Board of Health explored the options and barriers in the interactions between GPs and LGBT+ individuals. The report found, among other things, that patients felt they had to be experts in their own treatment, due to the lack of knowledge on transgender issues, among GPs and that they were limited and challenged by their their Civil Registration Number (CPR number) [2], which are some of the unique needs and challenges faced by TNB individuals when consulting their GP. However, the effect of these challenges was not illuminated, highlighting the need for further research on their impact.

Cultural safety (or cultural competence) has been identified as a means to address health inequities for minorities, including TNB individuals [5,10,11,13–15]. Cultural safety involves ongoing self-reflection and self-awareness, among health professionals, to acknowledge and identify one’s own biases, assumptions, stereotypes, and prejudices [13–15]. As part of practicing cultural safety, GPs create a ‘safe space’ for their patients, encouraging them to share intimate and sensitive information, which is crucial for addressing health inequities among TNB individuals [10–12,15]. In 2018, the Danish Health Authorities issued guidelines on healthcare related to gender identity, emphasising that, ‘The healthcare must be based on respect, responsiveness, inclusiveness and flexibility. […] the healthcare must be provided in a framework and atmosphere in which the person feels at ease’ [16]. This underscores the importance of GPs providing a ‘safe space’ for their TNB patients. A safe space is intended to be free from bias, conflict, criticism, or potentially threatening actions, ideas, or conversations, and is essential for patients to trust and open up to their GP [17–19].

This study examines the experiences of TNB individuals with primary care in general practice, with a focus on ‘safe spaces’. This study aims to identify the factors, according to Danish TNB individuals, that create, maintain, and disrupt a ‘safe space’ in general practice. A more profound comprehension of these patient experiences will provide a foundation for improving their healthcare and fostering their overall inclusion in society.

## Methods

### Settings

In Denmark, nearly the entire population is registered with a GP responsible for primary care. Assessment of gender identity and medical transitioning, such as hormone replacement therapy (HRT) and surgery, are exclusively carried out at the three Centres for Gender Identity (CKI). Transgender individuals who wish to transition medically must obtain a referral from their GP, positioning Danish GPs as gatekeepers in transgender care.

### Design, sampling, and participants

This study is a qualitative interview study, ideal for identifying experiences, expectations, thoughts, and feelings regarding what creates, upholds, and disrupts a ‘safe space’. An invitation to participate in interviews to understand personal experiences with general practitioners in Denmark was posted on the webpage of The National Association for Gay, Lesbian, Bisexual, and Transgender People in Denmark (LGBT+ Denmark), a political organisation, appealing to all ages, genders, and sexual orientations. Additionally, the invitation was shared in a closed Facebook group for nonbinary and transgender individuals with 839 members. This convenience sampling yielded a diverse group of participants, aged 20 to 43, comprising four transgender men, four transgender women, and four nonbinary individuals. In this study, ‘Transgender man’ also covers participants identifying as ‘trans masculine’ and ‘Nonbinary’ covers the gender identity ‘Gender fluid’. The participants’ characteristics are presented in Table 1. The sample had a broad regional representation, though there was a predominance of participants from Zealand. Inclusion criteria were that participants had to be 18 years or older, able to be interviewed in Danish or English, and have disclosed their gender identity to their GP. Twelve individuals responded and were included in the study. The sample primarily consisted of informants who had undergone gender-affirming healthcare or had been aware of their gender identity for over a year.

**Table 1.** Participant characteristics.

Participant*	Age	Assigned gender at birth	Gender identity
Leonora	34	Male	Transgender woman
Sally	29	Male	Transgender woman
Selma	25	Male	Transgender woman
Eddie	29	Female	Transgender man
David	27	Female	Transgender man
Jesse	24	Male	Nonbinary
Lee	28	Female	Nonbinary
Sia	43	Male	Nonbinary/ Gender fluid
Max	21	Female	Trans masculine
Sam	34	Female	Nonbinary
Olivia	20	Male	Transgender woman
Jay	35	Female	Transgender man

*All participant names are fictive.

### Interviews

Twelve semi-structured interviews were conducted by EVB between September and December 2022. An interview guide provided a flexible framework, covering areas such as gender identity, ‘safe space’, and GPs’ knowledge (Appendix 1). An independent review by LGBT+ Denmark assessed the interview guide for cultural sensitivity, and no modifications were recommended; they vouched for the cultural sensitivity of its content. Participants were informed about EVB’s background as a medical student and a member of the LGBT+ community. Ten individual interviews took place at the Research Unit for General Practice, University of Copenhagen, lasting between 40 and 90 min. Two interviews were conducted *via* Zoom due to geographical distances. One interview was conducted in English, while the rest were in Danish. All interviews were audiotaped and transcribed verbatim by the first author.

### Analysis

Data were analysed using Systematic Text Condensation, which involves four steps: 1) reading through all data and identifying themes, 2) moving from themes to codes by identifying and sorting meaning units, 3) condensing the data from codes to meaning, and 4) synthesising the data from condensation to description and concepts [20]. An example appears in Table 2. The primary analysis was conducted by the first author (EVB), with each step discussed with the qualitative researcher and last author (ADG). Both researchers read through the interviews, identifying themes by hand, coding, and sorting meaning units for the first four interviews.

**Table 2.** Shows the process for analysing our data using systematic text condensation.

Theme	Meaning units	Condensation	Synthesis
Unsafe experiences in General practice keep transgendered patients from seeking healthcare	I haven’t been at my new doctor yet because I don’t know what to expect. I moved recently and I had a negative experience with my old GP […] I don’t necessarily expect the worst, but I have to be prepared for a negative reaction. (Jay) Either they make fun of you or they just don’t take you seriously, and of course that makes you more cautious about consulting your doctor… and it shouldn’t be that way. (David) I would probably wait and consider if I could go somewhere else? Like around my GP at a public clinic or something? But if it was profoundly harmful to my health, I would bite the bullet and see my GP. (Lee) If I felt a tumour in my breast or my testicle hurt, or something… I’m not sure I would dare to see my GP… (Leonora)	Delayed onset from seeing new doctor and preparing oneself for negative reactions Not being taken seriously deters patients from consulting their doctor. Considering other options before consulting own GP Not daring to consult GP with problems	Previous negative experiences with GPs leads to healthcare avoidance

### Ethics

All participants provided informed consent both verbally and in writing. They were assured that their participation or non-participation would not affect their treatment at CKI or with their GP and that they could withdraw from the study at any time. Before each interview, participants were reassured that they could stop the interview or skip questions if they felt uncomfortable. Participants were guaranteed confidentiality and anonymity in the presentation of findings. The study complies with GDPR and was approved by the Danish Data Protection Agency. According to Danish research guidelines, no further approval was required for this type of study.

## Findings

### Unsafe experiences in general practice and heath care avoidance

Almost all participants reported feeling unsafe when consulting their GPs due to previous negative experiences related to their transgender identity. These experiences often deterred them from seeking healthcare when needed. Multiple participants reported postponing or avoiding visits to their GP altogether. In several cases, participants expressed reluctance to discuss their gender identity with their GP due to uncertainty about the doctor’s reaction. Some withheld information because they did not feel safe around their doctor. A 24-year-old nonbinary trans person explained;

There are some issues for which I perhaps should have consulted my GP or been honest about, but I have not done so because I did not feel safe or seen, and therefore I have omitted it.

Jesse, Nonbinary transperson, 24

Patients also stated that a negative experience with their GP could eventually lead them to seek a new one. Some participants emphasised that examinations for sexually transmitted diseases and other gender-specific examinations made them feel unsafe if the doctor was not attentive to the special needs of transgender patients. Consequently, some participants attended other clinics, such as AIDS-Fondet’s Checkpoint, a free-of-charge sexual-health clinic with an explicit focus on LGBT+ inclusivity.

Many participants felt unsafe when they had to explain themselves and advocate for their care. Some reported that when they approached their doctor with symptoms, the doctors would attribute these symptoms to HRT and, as a result, would not initiate any further examinations. These experiences reinforced the understanding that when discussing topics related to their gender identity with their GPs, doctors did not take them seriously.

It felt like not being taken seriously. It was odd that they blamed it on the HRT, because if someone with diabetes sees their doctor for a headache you do not just assume the medication is to blame. Of course all medication can have side effects, but you must investigate more to find the cause… look at the symptoms rather than guess?

David, Transgender man, 27

### A doctor who can contain their own attitudes and shortcomings

There was a consensus among participants that doctors’ personal attitudes towards transgenderism were always a factor to consider when seeking healthcare. Challenges were faced both when requesting a referral to specialized transgender care and during medical examinations.

It is unpleasant to be examined or to speak to someone who either shows disgust at having to examine you or seems excited because they find you interesting.

Jay, Transgender man, 35

Participants described medical procedures where doctors appeared nervous and insecure, which was an unpleasant experience for them. All participants emphasised that misunderstandings or a lack of knowledge could be remedied if the GP apologised. When GPs were upfront about their limitations, patients were generally very understanding. GPs who listened and met patients on their terms were considered supportive and capable of providing more holistic and adequate care. A nonbinary participant explained how they wished their GP had approached the conversation about gender identity.;

When I consult my doctor, I feel that he should at least… not necessarily understand fully, but to commiserate with what is being said and to be able to contain it.

Jesse, Nonbinary transperson, 24

Multiple participants expressed that they felt their GP was suspicious of them when the GP questioned their transgender identity or even argued that they were not transgender. This made the participants feel incapacitated. Some rationalised that the doctor’s motive was to try and help them, but they still thought that it was discriminatory to varying degrees. A transgender woman recounted her experience when she first came out to her GP and requested a referral to specialised transgender care;

His first comment was, ‘You are 32—why are you only coming now?’ […] Incapacitated. As if I did not know myself. As though I had made it up to be cool.

Leonora, Transgender woman, 34

### GPs making an effort to provide a ‘safer space’

During the interviews, it became apparent that the term ‘safer space’ was more appropriate than ‘safe space’, reflecting the understanding that while a space can be made safer, it cannot guarantee complete safety for every individual. When doctors made an effort to use patients’ chosen names and pronouns, or even inquired about them, the GP was perceived as providing a ‘safer space’. This effort made participants feel respected by their GP. A transgender man recounted how his new doctor handled the situation when he had not yet legally changed his name;

Even before I had legally changed my name, she updated my name in the system and asked which pronouns I preferred.

Eddie, Transgender man, 29

Several participants expressed a desire to feel prioritised by their physician and to have their specific needs for transgender care recognised. However, this was not the case for most participants. A transgender woman who had encountered significant resistance from her GP shared her experience;

To be seen as who I am—to have doctors recognise that trans people need treatment unique to their situation? That, I don’t feel they can manage at all.

Leonora, Transgender woman, 34

Participants reported that doctors who actively sought new options for their transgender patients and endeavoured to gain knowledge about this patient group were perceived as trans-friendly and as providing a ‘safer space’. Demonstrating interest and a willingness to explore options, even in the absence of knowledge about transgender care, was seen as a way for doctors to create a ‘safer space’. A transgender woman explained how her doctor gained knowledge from consultation to consultation, which made her feel safer with her GP;

When I went and finally asked for the referral to specialized trans care, then again, I felt that she was prepared […] She had options for me, she knew, she could recommend some things. […] I also felt empowered, that yes! I was lost, and now I have some tools, or some direction on what turn to take next in this.

Sia, Transgender woman, 43

Several participants noted that their doctor played an important role as a confidant during the early stages of their transition. The individual doctor could provide a ‘safer space’, laying the foundation for a good relationship. Participants described feeling safe with their doctors when the doctors listened to them and when they felt that the doctors believed them.

### GP’s lack of knowledge opposes a ‘safer space’

An overarching experience among all participants was the physicians’ lack of knowledge regarding transgender healthcare needs. Many participants reported that their doctors had limited to no knowledge about gender identity and how to communicate with and about transgender patients. This often meant that participants had to provide the necessary knowledge and vocabulary themselves. Misgendering and using incorrect pronouns were common examples of this issue. Participants felt that they had to create and uphold the space to have a conversation about gender identity, which made them feel as though they had to be able to treat themselves. A transgender man elaborated on his experiences with GPs regarding gender identity;

There was a huge lack of knowledge; they simply did not know how to care for a transgender patient. I had to tell them which blood tests mattered, how to give injections and how the system works for trans patients. Much of the time it was very uncomfortable.

Jay, Transgender man, 35

Multiple participants reported instances where doctors refused to assist them with issues related to gender identity. Participants speculated that this refusal stemmed from the doctors’ perceived lack of necessary knowledge, though some still felt it was discriminatory. Doctors often lacked understanding of seemingly fundamental aspects, such as obtaining an anamnesis, referring patients to specialised transgender care, available treatments for transgender patients, sexual health for transgender individuals, and baseline knowledge about HRT. A transgender woman recalled how her GP asked various misplaced questions when attempting to obtain an anamnesis;

I was sent back to the waiting area because he needed to look something up. He then asked me about my sexuality—’Are you into men, into women, into animals? What do you prefer? Your genitals—do they work? Are they malformed?

Leonora, Transgender woman, 34

Participants expressed a preference for their GP to ask questions rather than make assumptions. The distinction between asking overly personal questions that overstep boundaries and asking helpful questions lies in ensuring the patient understands the reason behind the inquiry. Questions that were clearly relevant to the matter at hand were generally not perceived as discriminatory and were broadly accepted, and in some cases, even appreciated. All participants preferred that doctors ask extensively about sexual health, particularly when testing for sexually transmitted diseases. This approach gave the impression that the questions were not based on assumed gender and sexuality, suggesting that the doctors had training in transgender healthcare. A transgender man, who consulted a doctor he was not registered with, explained why he felt this GP was able to uphold a ‘safer space’ while still asking the necessary questions to make a diagnosis;

He asks professional questions; he never asks things like ‘What about operations? What is your sexuality?’—those people tend to ask that. It felt professional, so when it was uncomfortable it was the ‘at-the-doctor’ kind of discomfort, not the ‘I am being violated’ kind. Ask to help the patient, not out of curiosity about something exotic

Jay, Transgender man, 35

### A binary way of thinking in the health care system

Participants felt that the healthcare system was very binary and not designed to accommodate trans people. They expressed the need for doctors to be aware of their own cisnormative assumptions (the belief that everyone is cisgender) and to avoid making assumptions about patients’ sexuality and bodily functions based on their gender identity. A 29-year-old transgender man recalled;

One time they misunderstood me on the phone when I said I was on hormones […]

the doctor thought I meant birth control.

Eddie, Transgender man, 29

Terms such as ‘man’ and ‘woman’ were perceived to be used in a very cisnormative way to describe certain traits, which participants found very unpleasant. A few patients expressed concerns that their doctor might not be able to assist them with issues related to their sexual health or refer them to gender-specific treatments if they changed their CPR number One participant, a nonbinary trans person who has endometriosis, explained how this concern had prevented them from making a legal sex change;

I want to make sure that I can get the same treatments as my biological gender, but if I didn’t need that, I probably would have changed it already…

Max, Nonbinary transperson, 21

All participants reported that doctors had made assumptions about them based on their gender, which triggered varying degrees of dysphoria. Not only was this considered unpleasant, but some participants also expressed concerns that it could be dangerous in a medical context, as assumptions about physiology and anatomy might be incorrect. A nonbinary person who had undergone a hysterectomy and was on HRT explained their fears regarding this issue;

My CPR number ends with an odd digit, so if I call them they will make assumptions about my anatomy and how my body functions. My biggest fear is that if I fall ill and am taken to hospital, depending on where I am hurting, no one will think, ‘Perhaps this is it, because this person used to have a uterus!

Sam, Nonbinary, 34

## Discussion

### Summary of findings

Almost all participants felt unsafe with their GPs due to previous negative experiences related to their transgender identity, leading to healthcare avoidance. Participants were reluctant to discuss their gender identity with their GP, fearing negative reactions or a lack of understanding. Negative experiences, particularly during gender-specific examinations, led some participants to seek care at alternative clinics. When doctors uses patients’ chosen names and pronouns, it contributed to providing a ‘safer space’. A completely ‘safe space’ was considered unrealistic by participants, who preferred the term ‘safer space’. Participants appreciated GPs who showed interest in learning about transgender care and who were upfront about their limitations.

Supportive GPs who listened and met patients on their terms were seen as providing more holistic and adequate care. A significant issue was the lack of knowledge among GPs regarding transgender healthcare needs. Participants often had to educate their GPs, which included correcting instances of misgendering and inappropriate use of pronouns. Some participants felt that their GPs’ lack of knowledge led to discriminatory practices and inadequate care, which challenged a ‘safer space’. Participants felt that the healthcare system was very binary and not created to fit trans people. They expressed the need for doctors to stop making assumptions about patients’ sexuality and bodily functions on their gender identity.

### Healthcare avoidance

This study suggests that previous negative experiences with primary care undermine TNB patients’ sense of feeling safe when consulting their GP, disrupting disclosure of gender identity related issues and prompting healthcare avoidance. A large Ontario respondent‑driven sample found 47.7% of transmasculine and 54.5% of transfeminine patients with a regular family physician reported discomfort discussing trans‑related health issues [9]. Such findings have been reported in Canada, New Zealand, and Sweden, and our study adds that this issue also affects

TNB individuals in Denmark, suggesting that this is a widespread issue for transgender people regardless of geography [9–11,21]. Gender‑specific issues were a commonly reported source of negative experiences. Participants in our study reported switching to alternative clinics rather than their registered GP. Similarly, in a qualitative Australian study, several respondents described seeking different clinics when faced with disrespect, misgendering, or inappropriate sexual‑health examinations [22]. While care for sexual health may be accessible at other clinics, TNB individuals rely on their GP for treatment of many different conditions. This highlights the higher level of unmet health needs experienced by many TNB individuals [1–3,9–11].

### A ‘safer space’

In our study, participants identified several issues that challenged the ‘safer space’ and impacted their inclination to utilising healthcare services. Many participants felt unsafe when they had to advocate for their care. Some reported not receiving medical examinations when presenting symptoms, as doctors attributed these symptoms to HRT, while others described experiences of overt discrimination. A 2019 literature review highlights the importance of GPs applying cultural safety, as a means of addressing the health inequities experienced by patients from minority groups, which includes creating a ‘safe space’ for patients from minority groups [15]. A Canadian, qualitative study concluded that providing safe spaces for transgender patients could increase the likelihood of them accessing healthcare when needed [10].

To our knowledge, the concept of providing a ‘safer space’ has not been explicitly articulated in the Danish context concerning transgender patients. Our study provides new insights into the lived experiences of Danish TNB individuals and suggests areas for improvement. Participants agreed that GPs who listened and met patients on their terms created a ‘safer space’: using chosen names and pronouns, acknowledging and apologising for mistakes, being upfront about their limitations, and actively seeking information or alternative options for transgender care. These behaviors significantly increased trust and encouraged continued use of primary care, underscoring the importance of their actions. The Danish Health Authorities recommended that; ‘[…] the healthcare must be provided in a framework and atmosphere in which the person feels at ease’ [16], which reduces exposure to stigma and minority stress and enhances social support and resilience—effects associated with improved mental health outcomes for LGBT+ people and consistent with the concept of a ‘safe space’ [18,19]. It could be beneficial to incorporate ‘safer space’ as a technical term in medical language, facilitating education on the matter. This could eventually lead to not only transgender patients feeling safer when utilising healthcare services but also other minority groups [2,10–12,21].

### Asking the right questions

Some GPs were perceived by informants to ask overly personal or invasive questions that were irrelevant to the matter at hand during consultations with TNB individuals. A systematic review from 2024 found that, such non‑clinically relevant questioning by some general practitioners was reported to cause feelings of exposure and mistrust among transgender and nonbinary patients [12], and this issue is also documented previously [23–25]. Respondents in our study perceived such questions as stemming from the GPs’ unprofessional curiosity, which was seen as a form of discrimination. Some participants noted that it made them feel like they were an exotic experience rather than a human being.

Our findings indicate that patients preferred their GP to ask questions during a consultation rather than make assumptions. The key element in asking questions in a respectful way was ensuring that the patient understood why the GP was asking them. Questions that were clearly relevant to the matter at hand were generally not perceived as discriminatory and were largely tolerated. This explanation of how GPs can ask questions without violating patients’ boundaries is, to our knowledge, not previously illuminated. It contributes to the existing knowledge on how GPs can maintain a ‘safer space’ for trans individuals during medical examinations and consultations.

### Strengths and limitations

We applied the information-power framework to evaluate strengths and limitations, attending to study aim, sample specificity, and interview quality [26]. The study’s focused aim—to identify the factors, according to Danish TNB individuals, that create, maintain, and disrupt a ‘safe space’ in general practice.—increased the study’s information yield by concentrating data collection on a clearly defined phenomenon. Recruitment through both LGBT+ Denmark and a broadly accessible Facebook group, using convenience sampling, captured substantial variation across the trans spectrum and ensured that all participants had direct experience discussing gender identity with their GP, producing a rich dataset. The nature and purpose of a qualitative semi-structured interview were ideally suited to exploring a research question about individuals’ experiences with safe spaces in primary care. This approach allowed flexibility to ask further questions when a subject of interest arose, enabling adjustments to the interview guide over time. The interview dialogue was strong. The first author and interviewer possessed above-average knowledge about LGBT+ individuals and accessing healthcare, as he is part of the LGBT+ community. Participants were informed of this, and many noted that it was an important factor in establishing trust during the interview. It is possible that participants were more likely to share their experiences freely and provide information which they might not have shared with a non-LGBT+ interviewer, due to a mutual understanding. Consequently, the interview dialogue was both rich and meaningful. To ensure that crucial information was not missed or omitted due to implicit understanding, ADG listened in on the first few interviews to establish a meta-position, and adjustments were made accordingly. Given the study’s narrow focus, participant specificity, high-quality interviews, and rigorous analysis, we judge the dataset to be sufficiently information-rich to address the research question.

This study has some limitations regarding transferability. Online recruitment is likely to have favored younger, digitally active individuals and, *via* LGBT+ Denmark, those more engaged in community networks or activism; recruitment *via* Facebook broadened reach but did not eliminate selection bias. Self-selection may have over-represented participants with negative healthcare experiences or those with the capacity to participate, potentially skewing findings.

## Conclusion

This study provides insight into the lived experiences of Danish TNB people in general practice, showing that primary care comprises both discriminatory and affirming practices. Participants report that negative encounters commonly lead to delayed or avoided help‑seeking, while respectful behaviours—such as using chosen names and pronouns, listening attentively, and demonstrating knowledge of transgender care—promote a sense of safety.

Integrating ‘safer space’ as a technical medical term may facilitate education on the matter, potentially leading to transgender patients feeling safer when utilising healthcare services.

### Implications for practice and research

This study provides the basis for two key points for GPs.

First, simple measures such as being upfront about one’s limitations, apologising when making a mistake, showing interest and willingness in exploring treatment options, gaining knowledge about the patient group, and using patients’ chosen names and pronouns are all ways, according to our informants, that GPs can create a ‘safer space’ for trans identities and thus potentially increase access to health care.

Second, transgender patients prefer their doctors to ask questions rather than make assumptions about, for example, assumed gender, sexual activity, and bodily functions. However, it is crucial that GPs ask only when it is relevant to the matter at hand and ensure they provide proper information about why they are asking these questions, thereby avoiding overstepping patients’ boundaries.

Further research should investigate physicians’ experiences in providing care for transgender and nonbinary individuals to better understand the barriers they encounter. Furthermore, the findings from this study could serve as a foundation for developing a study to determine whether these themes are applicable to a broader sample of Danish transgender and nonbinary (TNB) individuals. Collaboration between relevant stakeholders to ensure that this research is conducted in a culturally safe manner is of utmost importance. Collectively, these studies could inform an intervention study to further explore whether it is possible to make general practice a ‘safer space’ for TNB individuals and thereby reduce the health inequities they experience.

## Supplementary Material

Glossary of Terms .docx

Interview guide_final.docx
