# Supplementary material for: The experiences of transgender and nonbinary individuals in general practice in Denmark, with a focus on ‘safer space’
Source: Scand J Prim Health Care. 2025 Dec 15;44(1):2599986. doi: 10.1080/02813432.2025.2599986 (PMC12710257; doi:10.1080/02813432.2025.2599986)
Supplement: Glossary of Terms .docx [file IPRI_A_2599986_SM4089.docx]

**Glossary of Terms & Abbreviations**

**Cisgender**: A person whose gender identity and gender expression align with the sex they were assigned at birth. (LGBT+ Denmark Dictionary: Cisgender) (https://lgbt.dk/ordbog/ciskoennet/)

**Cisnormativity:** The assumption and norm that everyone is cisgender. This norm renders invisible and marginalises those who deviate from it and are not cisgender. (LGBT+ Denmark Dictionary: Cisnormativity) (https://lgbt.dk/ordbog/cisnormativitet/)

**CKI**: Centres for Gender Identity. The specialised hospital units for trans healthcare in Denmark.

**GAHC**: Gender-Affirming Healthcare. Medical care that supports a person's gender identity, including hormone therapy, surgeries, and mental health services.

**GP**: General Practitioner.

**HRT**: Hormone Replacement Therapy. A treatment using hormones to align physical traits with gender identity.

**LGBT+ :** An abbreviation for Lesbian, Gay, Bisexual, Transgender, and other identities that do not fall under cisgender and heterosexual. The "+" indicates the inclusion of additional gender and sexual identities beyond those listed. (LGBT+ Denmark Dictionary: LGBT+) (<https://lgbt.dk/ordbog/lgbt/> )

**Nonbinary:** A gender identity that describes individuals who do not identify as either male or female, which are the two binary genders in the binary gender system, and therefore do not identify with the sex they were assigned at birth. (LGBT+ Denmark Dictionary: Nonbinary) (<https://lgbt.dk/ordbog/non-binaer-koensidentitet/> )

**Safespace/safer space :** This term refers to an environment where individuals can relax and express themselves fully and freely without fear of feeling uncomfortable, unwelcome, or unsafe due to their identity. (LGBT+ Denmark Dictionary: Safe Space) (https://lgbt.dk/ordbog/safe-space/)

**Trans woman:** A woman who was assigned male at birth. (LGBT+ Denmark Dictionary: Trans Woman) (<https://lgbt.dk/ordbog/transkvinde/> )

**Transgender/Trans Person:** An umbrella term for individuals whose gender identity does not fully align with the sex they were assigned at birth. (LGBT+ Denmark Dictionary: Transgender) (https://lgbt.dk/ordbog/transkoennet/)

**Trans man:** Trans Man: A man who was assigned female at birth. (LGBT+ Denmark Dictionary: Trans Man) (https://lgbt.dk/ordbog/transmand/)

**TNB**: Transgender and Nonbinary
