# Supplementary material for: The experiences of transgender and nonbinary individuals in general practice in Denmark, with a focus on ‘safer space’
Source: Scand J Prim Health Care. 2025 Dec 15;44(1):2599986. doi: 10.1080/02813432.2025.2599986 (PMC12710257; doi:10.1080/02813432.2025.2599986)
Supplement: Interview guide_final.docx [file IPRI_A_2599986_SM4088.docx]

**Interview guide**

**Project title:**
*“Danish Transgender and Nonbinary individuals’ experience in general practice with a focus on ‘safer space’. A qualitative interview study”*

| **Purpose** | How do transgender patients perceive their interactions with general practice, and what factors contribute to these encounters being perceived as safer spaces? |
| --- | --- |
| **Anonymity** | The interview will be recorded using a dictaphone and subsequently transcribed verbatim. Your participation will remain completely anonymous, with only my supervisor and myself having access to the interview during the analysis process. Quotes from the interview may be included in the final article, but all identifying information will be removed to ensure your anonymity is preserved. |
| **Practical** | The interview will last approximately 60 minutes.  Some of the questions may seem unusual in a typical conversation, but it is crucial in an interview setting to ensure clarity and avoid any ambiguity. Additionally, it is important that your responses are conveyed in your own words, rather than my interpretations.  Do you have any questions regarding this part of the interview? |
| **About me** | I am a medical student employed as a research assistant at the Research Unit for General Practice, University of Copenhagen. A part of the LGBT+ community.  I will be conducting the interview, and I am genuinely interested in hearing all of your thoughts and experiences. If there is anything on your mind that I do not specifically ask about, please feel free to share it. |
| **Structure** | We start out with some questions about your gender identity and background.  Subsequently, the questions will focus on your experiences with your general practitioner, including interactions with other personnel you may encounter at the doctor's office.  Finally, I will ask you about the concept of a safer space, how one can create such an environment, and your perceptions of it. |
| **Before we begin..** | Lastly, I want to remind you that you can withdraw your consent at any time. You also have the option to request a break or stop the interview at any point. We can always resume on another day if needed.  There are no right or wrong answers in this interview, as I am interested in hearing your experiences and perspectives.  Please feel free to stop me at any given moment if you feel uncomfortable or have any comments.  Do you have any questions before we begin? |
| **Background** | How old are you?   In what area do you live?  How do you identify?  Which pronouns do you use? |
| **Warm-up Questions** | Could you describe a visit to your doctor's office in chronological order?  Additionally, could you provide an example of a situation where you felt either safe or unsafe while consulting your general practitioner? |
| **Gender identity** | How would you like your doctor to approach you?  What expectations do you have of your doctor regarding your gender identity? Can you describe a situation where your expectations were either met or where you felt unacknowledged? Why or why not?  Can you describe a situation during a consultation with your doctor where gender identity was the primary focus?  How did you discuss your gender identity with your doctor? How did you experience this interaction?  Please describe a situation where you considered bringing up your gender identity. Why did you decide to do so or not? What thoughts did you have?  Have you ever experienced your doctor using language that you found offensive or discriminatory during a consultation? How did this make you feel? |
| **Safe space** | Now we will transition to a slightly different topic. I would like to discuss the concept of general practice as a safer space.  What does a safer space mean to you? What comes to mind when I mention 'safer space' in the context of general practice? Could you elaborate on this and provide examples?  Can you describe a positive experience you have had with your general practitioner?  Have you ever experienced discrimination due to your gender identity by your general practitioner or other personnel at your doctor's office? Please provide examples. What could your doctor have done differently? |
| **Knowledge & improvement** | Do you feel that your doctor possesses the necessary knowledge to advise and treat you optimally as a transgender person? Can you provide examples?  Do you believe that your doctor uses appropriate vocabulary (e.g., pronouns, terminology) when communicating with transgender patients, ensuring they feel acknowledged and safe?  How can we ensure that general practitioners are better prepared to meet the needs of transgender individuals and accommodate their specific requirements?  Do you have any additional suggestions for improvement? |
| **General practice as a whole** | How do you perceive your general practice as a whole?  Do you feel represented in your doctor's office? Consider aspects such as bathrooms, other personnel, and the waiting area. |
| **Concluding Questions** | Is there anything else you would like to discuss that we haven't covered yet?  Were there any questions that you found unusual or out of place? Is there anything you are curious about? |
